# A descriptive study of human papilloma virus in upper aero-digestive squamous cell carcinoma at Uganda cancer institute assessed by P16 immunohistochemistry

**DOI:** 10.1186/s41199-020-00057-3

**Published:** 2020-08-27

**Authors:** Fiona Kabagenyi, Jeff Otiti, Justine Namwagala, Adriane Kamulegeya, Sam Kalungi

**Affiliations:** 1 Department of Ear, Nose and Throat, College of Health Sciences, Makerere University, P.O. Box 7072, Kampala, Uganda; 2 Department of Surgery, Uganda Cancer Institute, P.O. Box 3935, Kampala, Uganda; 3 Department of Oro-maxillofacial Surgery, College of Health Sciences, Makerere University, P.O. Box 7072, Kampala, Uganda; 4 Department of Pathology, Mulago National Referral Hospital, P.O. Box 7272, Kampala, Uganda

**Keywords:** Human papilloma virus, Upper aero-digestive squamous cell carcinoma, Head and neck cancer, Uganda Cancer institute, p16 immunochemistry

## Abstract

**Background:**

Cancer burden in sub-Saharan Africa is on the rise with one-third of cancers estimated to be caused by infectious agents. Head and neck squamous cell cancer (HNSCC) is the sixth most common malignancy in sub-Saharan Africa and includes tumors in the Upper Aero-digestive Tract (UADT). The established risk factors are tobacco and alcohol exposure with a recent recognition of the role of Human Papilloma Virus (HPV). The HPV related HNC is seen predominantly in the oropharynx, presents at a younger age and has a better prognosis. With a rapidly increasing incidence of these cancers in the developed world, it was important to study HPV in HNC in Uganda. The HPV can be detected using P16 immunohistochemistry as a surrogate marker thus making it suitable for screening. The study aimed at establishing the presence of HPV and the commonly affected sites in UADT squamous cell carcinoma (SCC) at Uganda Cancer Institute (UCI) using P16 immunohistochemistry.

**Methodology:**

This was a cross sectional study in which 59 patients with histologically proven SCC from the oral cavity, oropharynx, larynx and hypopharynx were recruited. These patients’ demographics and clinical data were collected. Tissue sections from retrieved histology samples were stained by Haematoxylin and Eosin to reconfirm SCC. Subsequently, P16 expression was determined using P16 immunohistochemistry.

**Results:**

Seventy-one patients were enrolled and 59 patients with confirmed SCC of the sites of interest were analyzed. The majority (79.7%) of the participants were male and over 50 years. 59.3% were tobacco smokers, 66.1% used alcohol, 52.2% used both. Only 27.1% used none of the substances. Only 27.1% of the participants were HIV positive. Most of the tumors were in the larynx (37.3%) and 64.4% were overall TNM stage 4. The overall prevalence of HPV in UADT SCC at UCI was 20.3, 95%CI 10.9–32.8. The oropharynx had the highest prevalence (30.8%).

**Conclusion:**

The prevalence of HPV in UADT SCC at UCI is significant at 20.3%. The most affected site, is the oropharynx. Vigilant HPV screening of these sites with confirmation where possible is recommended.

## Background

Head and neck carcinoma (HNC) ranks sixth among the most common cancers seen worldwide [1]. The commonest histological type is squamous cell carcinoma (SCC) accounting for more than 90% of HNC [2]. Globally and locally, more than half of these cancers arise from the oral cavity/ oropharynx [1, 3].

The etiology of head and neck squamous cell carcinomas (HNSCC) has mainly been attributed to tobacco and alcohol consumption [4]. However the increasing incidence of head and neck squamous cell carcinomas (HNSCC) seen in the developed world has been attributed to the Human Papilloma Virus (HPV) [5, 6]. It is noteworthy that HPV is the causative agent of cervical cancer for which the burden is extremely high throughout sub-Saharan Africa [7].. Therefore we believe that the HPV is very likely to play a key role in other cancers within this subcontinent. However, the contribution of HPV to HNSCC in Uganda is largely unknown. Twenty five percent of all HNSCC are HPV positive [8]. The commonest site for HPV positive HNSCC is the oropharynx with frequencies ranging from 39 to 56% in the developed world and 13% in the rest of the world [9]. Other sites involved are the oral cavity, hypopharynx and larynx [1, 10, 11].

High risk HPV causes dysregulation of the cell cycle at the molecular level. The HPV produces onco-proteins that affect three tumor suppressor genes (p53, Rb and p16) in the host cell. When the HPV onco-protein E7 binds to the retinoblastoma gene in the host cell, it consequently releases the inhibition of P16 gene. This in turn causes an increased expression of p16 protein which can be detected by immunohistochemistry. P16 is currently used as a surrogate marker for HPV [12, 13]. Prior studies have used varying methodologies to detect HPV [immunohistochemistry (IHC), polymerase chain reaction (PCR), or a combination], often without detailed characterisation of anatomic site or simultaneous evaluation of other risk factors including HIV infection [14–17]. We therefore sought to determine the presence of HPV using p16 IHC among histologically confirmed HNSCC in cases at Uganda Cancer Institute.

## Methods

This study was approved by Makerere University’s School of Medicine Research and Ethics Committee and that of Uganda Cancer Institute. This was a cross sectional study conducted from October 2018 to May 2019.

### Study site

The study was conducted at Uganda Cancer Institute (UCI), a public, specialised tertiary care center for cancer treatment, research and training. It is located in the urban centre of Uganda’s Capital City Kampala. It serves the 42 million Ugandan population in addition to referrals from neighbouring countries like South Sudan and DRC. Approximately 200 cancer patients are seen daily with an average of 8 new HN cases. 400 patients with HNC per year are seen on referral basis with their histologically confirmed biopsies (from multiple laboratories both institutional and private) for staging and treatment planning in a tumor board setting.

### Study participants

For this study, upper aero-digestive tract (UADT) SCC comprised of the oral cavity, oropharynx, hypopharynx and larynx only. We recruited all patients that consented to participating in the study. On the other hand, patients with history of prior radiotherapy, those whose tissue blocks could not be accessed from the pathology labs and those whose retrieved blocks showed no malignancy or had insufficient tissue for histological analysis were excluded.

We captured age, gender, education level, occupation, socioeconomic status, history of smoking and alcohol ingestion, HIV status (documented evidence), sexual history and the Tumor, Node, Metastasis (TNM) stage of the patient (American Joint Committee on Cancer AJCC 7th edition). TNM staging was captured after it was agreed upon in the HN tumor board. Our dependant variable was the HPV status. For purposes of this study, it was assumed that a p16 positive stain meant HPV positive and negative staining for p16 expression meant HPV negative.

### Study procedures

We used the Kish Leslie (1965) formula for sample size estimation [using the study in Sudan, [15], adjusted to our local context using the finite correction factor and aimed at having a minimum 70 patients. Consecutive sampling was used to attain the sample size**.**

During the study period, patients with result slips showing histologically confirmed UADT SCC were interviewed. Their tissue blocks were retrieved from accessible pathology labs. The tissue blocks were then collected at the UCI pathology laboratory for histological re-confirmation followed by p16 IHC.

### Laboratory methods

#### Tissue processing and H&E

Retrieved Formalin Fixed Paraffin Embedded tissues were trimmed into 3–5 μm’ thickness and prepared serially for routine H&E staining method by use of standard operating procedure (SOP) (Additional file 2) by the technician. The H&E tissue slides were reviewed by technician, investigators and results confirmed with the assistance of two pathologists and entered into the respective data collection form. Those that did not show malignancy were excluded from further laboratory testing for p16 IHC.

#### Tissue processing and immunohistochemistry (IHC)

Consequently, tissue slides for IHC were prepared serially by first trimming tissue into 5 μm thick following the SOP as is outlined in the protocol (Additional file 3) for demonstration of p16 protein status. The protocol as provided by the Ventana Benchmark XT machine was used for immune staining of histological sections for p16 expression. Both positive and negative controls were stained in parallel [15].

#### Scoring of p16 gene expression immuno-staining

P16 protein expression was considered positive when tumor cells stained brown with different colour intensity (Additional file 4). Positive and negative controls were used to aid the scoring. Slides were reviewed by the technician and by two pathologists. The results were then entered into the respective data collection form when at least two pathologists were in agreement (Additional file 4). Positive results were reported with regard to site of staining, intensity of staining (> 70%) and percentage of tumor cells staining (Additional file 3) [18]. Samples for IHC were run with positive (known HPV positive carcinoma cervix) and negative controls (omission of antibody during the staining).

### Data analysis

Participant characteristics were expressed as categorical and/or continuous variables. Continuous variables were expressed as means and standard deviations while categorical data was expressed as frequencies with their respective proportions. The main outcome of this study was prevalence of HPV, which was presented as frequencies and proportions.

The data was analyzed using STATA 13.0. To determine the overall prevalence of HPV in UADT SCC at UCI, the number of participants that tested positive for HPV was divided by the total number of participants in the study. For site-specific prevalence of HPV in UADT SCC at UCI was determined by dividing the number of HPV positive by the total number of that site. A *p*-value of 0.05 or less was considered statistically significant. The analysis was done by the study biostatistician. We stratified our data according to P16 status to secondarily compared the results with sociodemographic and behavioural characteristics.

## Results

**Fig. 1:**
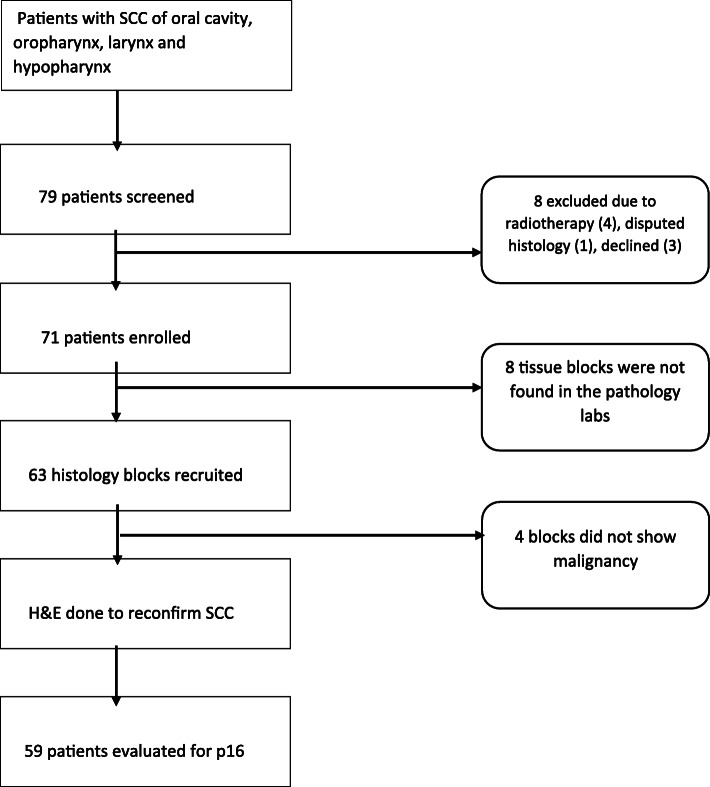
Study flow. All patients with histologically proven squamous cell carcinoma from the oral cavity, oropharynx, larynx and hypo-pharynx at UCI were consecutively sampled, then recruited and ultimately 59 were evaluated for P16

A total of 79 patients were screened and 71 were enrolled for the study. Fifty nine patients were finally analysed as shown in Fig. 1.

**Fig. 2:**
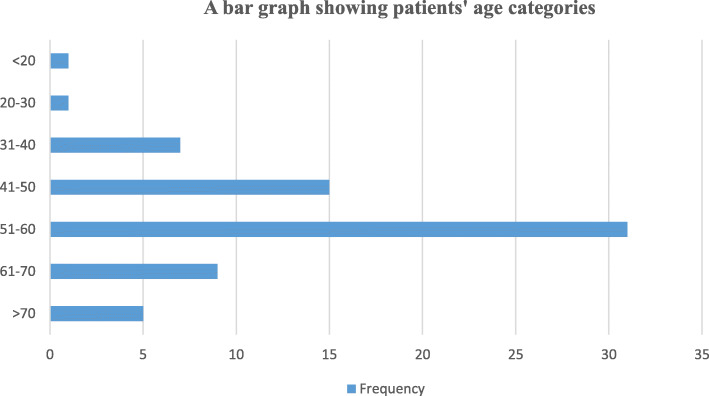
A bar graph showing patients’ age categories

Regarding participant’s socio demographic characteristics, all were of the black race with the majority of the participants being male (47/59) (Table 1). The median age was 54 +/− 12 with the youngest being 15 years and the oldest being 81 years. The most common age group was 51–60 years as shown in Fig. 2.

**Table 1:** Participants’ demographics and behavioural characteristics in relation to P16 status (denoting HPV status)

Variable	P16 status (denoting HPV)		*P* value
	Positive n (%)	Negative n (%)	
**Sex**			0.695
Male	9 (19.2)	38 (80.8)	
Female	3 (25)	9 (75)	
**Age**			0.175
≤ 40	0 (0.0)	9 (19.2)	
41–50	2 (16.7)	13 (27.7)	
51–60	7 (58.3)	14 (29.8)	
> 60	3 (25.0)	11 (23.4)	
**Substance use**			0.649
None	5 (31.3)	11 (68.7)	
Tobacco use only	1 (20)	4 (80)	
Alcohol use only	1 (14.3)	6 (85.7)	
Both alcohol and tobacco use	5 (16.1)	26 (83.9)	
**Sexual partners**			1
None / one only	5 (20)	20 (80)	
More than one	7 (20.6)	27 (79.4)	
**Oral sex**			0.906
Declined	2 (18.2)	9 (81.8)	
No	8 (22.9)	27 (77.1)	
Yes	2 (15.4)	11 (84.6)	
**HIV status**			0.718
Positive	4 (25)	12 (75)	
Negative	8 (18.6)	35 (81.4)	

Participants’ demographics and behavior characteristics were evaluated using proportions and frequencies

**Fig. 3:**
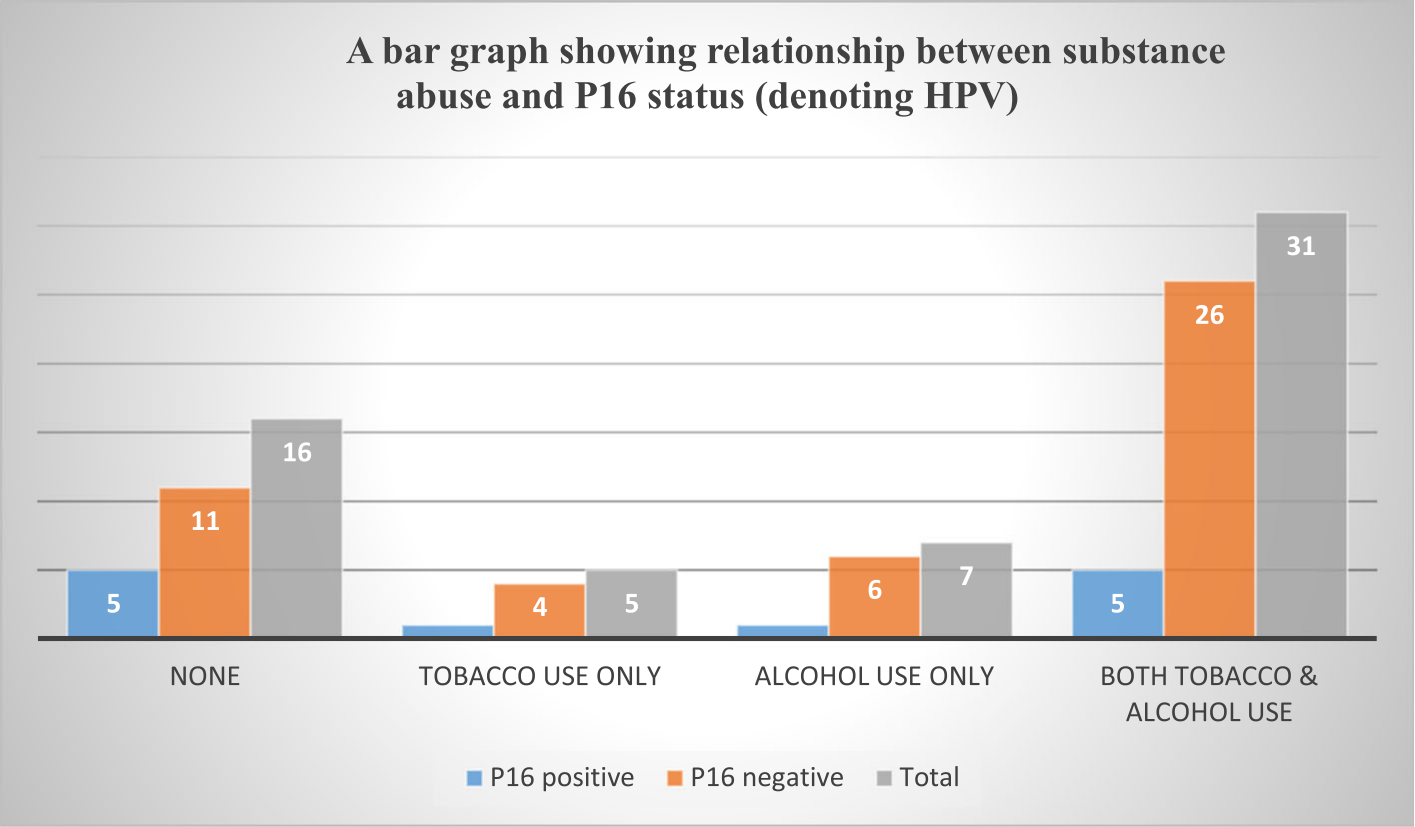
A bar graph showing relationship between substance abuse and P16 status (denoting HPV)

Most of the participants used tobacco (59.3%), 66.1% used alcohol and 52.5% used both tobacco and alcohol. The HIV positive participants constituted 27.1% (Fig. 3, Table 1).

**Fig. 4:**
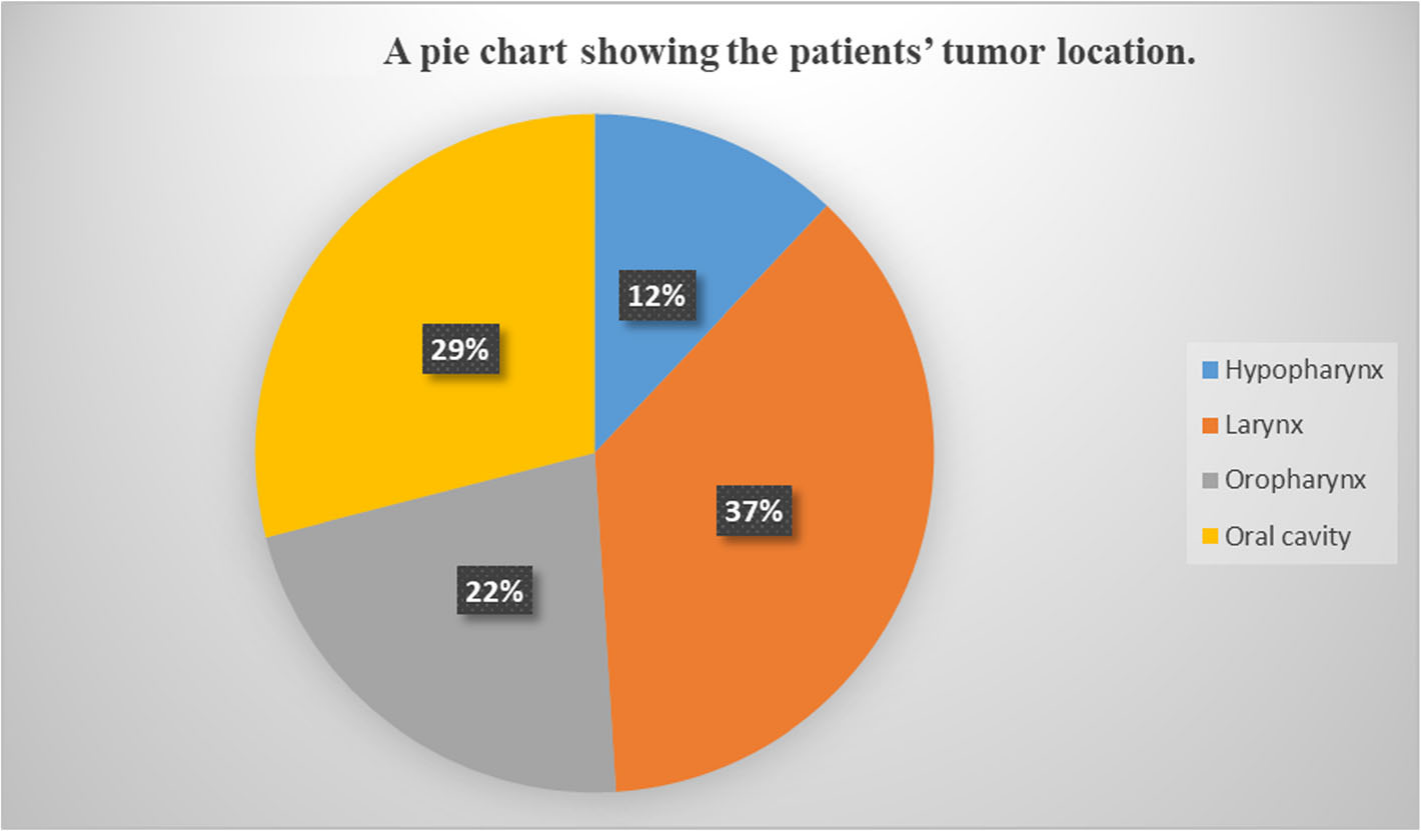
A pie chart showing the patients’ tumors location

**Fig. 5:**
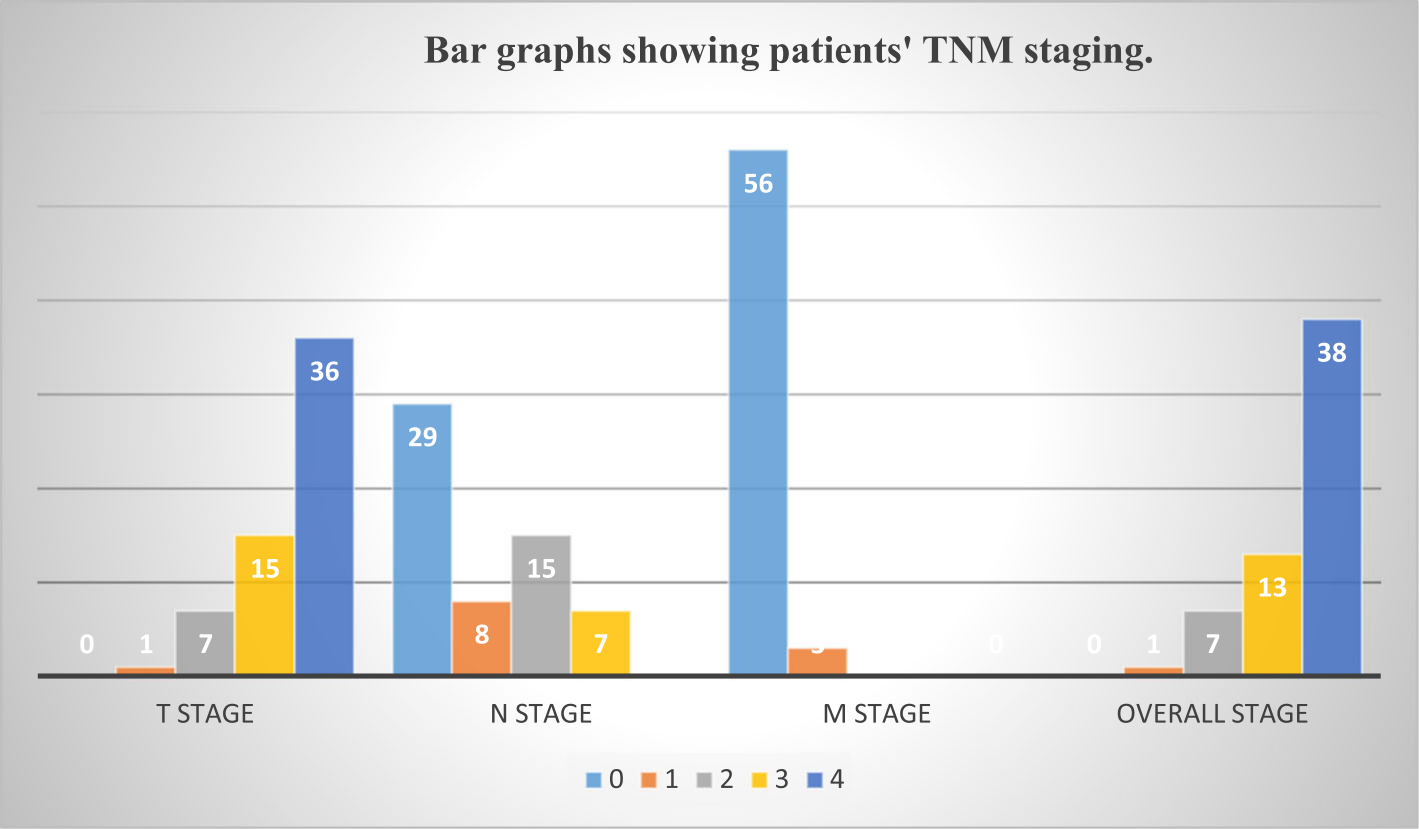
Bar graphs showing patients’ TNM staging

For participants’ tumor characteristics, most of the tumors were in the larynx (37.3%) followed by the oral cavity as shown in Fig. 4. In relation to the TNM staging, 61% were T stage 4, 49.1% had N0 stage while 50.9% had positive nodal stage with only 5.1% having distant metastases. The commonest overall stage was stage 4 (64.4%) as shown in Fig. 5.

The overall prevalence of HPV in UADT SCC at UCI using P16 IHC was 20.3, 95% Confidence interval (CI) of 10.9–32.8.

The site specific prevalence of HPV in UADT at UCI; the oropharynx had the highest prevalence (30.8%), followed by the oral cavity (29.4%) as summarized in the Table 2.

**Table 2:** Site- specific prevalence of HPV in UADT at UCI

Tumor site	P16 expression denoting HPV status	
	Positive n (%)	Negative n (%)
Hypo pharynx	1 (14.3)	6 (85.7)
Larynx	2 (9.1)	20 (90.9)
Oral cavity	5 (29.4)	12 (70.6)
Oropharynx	4 (30.8)	9 (69.2)

The patients with positive p16 were analyzed and overall prevalence of HPV in UADT SCC at UCI evaluated as a percentage of all study participants while site-specific prevalence of HPV in UADT SCC evaluated as a percentage of the total number of that site

## Discussion

### Overall HPV prevalence

The overall prevalence of HPV in UADT SCC using p16 IHC in this study was significant at 20.3%, lower than the global incidence of 25% but similar to some studies in Sub Saharan Africa (SSA) [8, 15, 16]. The leading site affected by HPV in our study was the oropharynx, in agreement with most studies inclusive of SSA [15–17]. We stratified our participants into P16 positive (denoting HPV positive) and P16 negative (denoting HPV negative) groups for comparison with gender, age, substance use and sexual behaviour but there was no statistical significance probably due to our small number.

The commonest detection methods for HPV in HNC worldwide are PCR and ISH [19] However, in studies from SSA, P16 IHC (as a surrogate marker for HPV) is greatly utilised followed by PCR, or both PCR and P16 IHC [14, 15, 17, 20]. Of the studies that utilised p16IHC, Ahmed et al. in Sudan found an overall HPV prevalence of 20.7% while Faggons et al. in Malawi found a 17% prevalence [15, 16]. These findings agree with our study. Of those that used both P16 IHC and PCR, Ndiaye et al. in Senegal found no positive stains for P16 and a 3.4% HPV prevalence on PCR while Sekee et al. in South Africa found a prevalence of 19.6% using P16 IHC that dropped to 6.3% using PCR [14, 17]. The variation seen with the different methods of HPV testing suggests the possibility of a different prevalence if we subject our study to PCR. In a systemic review by Larsen et al., P16 staining was found to be more predictive of HPV positivity (confirmed by PCR or ISH) when the cut off was > 70% of cytoplasmic and nuclear staining, a cut-off that was integrated in our study [18].

### Site-specific HPV prevalence

The sites of interest in our study were the oral cavity, oropharynx, larynx and hypopharynx. Several studies from SSA often combine other sites from the head and neck like the esophagus, paranasal sinuses, nasopharynx and even tumours of unknown origin. In addition, the distinction between the oral cavity and oropharynx is seldom made and the pharyngeal subsites are not grouped into naso/oro or hypopharyngeal sites [14–16]. This made it hard for consistent comparison of our results within SSA. The inclusion of subsites was beyond the scope of our study.

### Oropharynx

The oropharynx(OP) had the highest HPV prevalence (30.8%) compared to the non- oropharyngeal (NOP) sites in agreement with most studies [5, 7, 9]. In a meta-analysis, the trend of HPV related OPSCC (from before 2000 to 2009) was shown to rise in incidence while the NOP were stable over time, and much lower in prevalence. Africa had no data on HPV in these cancers then and to date, very little is documented. This meta-analysis found an overall HPV prevalence in OPC of 47.7% which is also higher than our finding [21]. An intercontinental case control study that utilised PCR showed the HPV contribution of 18.3%, almost doubled in our findings [22]. This may explain the difference seen with our study that had small numbers and used P16 IHC.

In SSA, Ahmed et al. made no distinction of oropharynx or hypopharynx [15]. Faggons et al. aggregated the oral cavity with the oropharynx [16]. We can only compare with Sekee et al., who found a prevalence of 25%, almost similar to our findings possibly due to similar methodology and population [17].

In the developed world, higher proportions of 39–56% are seen compared to our study [9]. Great strides in research involving oropharyngeal SCC have been made in these regions. Studies have found that HPV is common in patients aged 40s and 50s, never smokers or with reduced tobacco exposure with a higher number of lifetime oral sex partners and or vaginal sex partners [5, 8, 22–24]. Other studies also link the HPV-positive head and neck cancers to higher socioeconomic status [25, 26]. In a meta -analysis by Ragin et al., HPV represented a major cause of OPSCC among White patients compared to Blacks and Asians who had lower rates [19]. A study comparing the oral sexual behaviour, HPV infection and OPSCC trends by gender, age and race found that the White male of a younger cohort engaged in more oral sexual behaviour and was also at a higher risk of having HPV infection and OPSCC compared to their Black counterpart [27]. Therefore, the variance seen with our study may be due the lower socioeconomic status of our subjects, black race and the differing substance use and sexual patterns.

### Non- oropharyngeal sites

The prevalence of HPV in the oral cavity (29.4%) in our study was slightly higher than that of Ahmed et al. (22%) [15]. This similarity may be explained by the proximity of the geographical locations of Uganda and Sudan. The prevalence of HPV in the oral cavity was eight times higher in our study when compared to that of Herrero et al. (3.9%) [22]. This discrepancy may also be attributed to the large numbers used in this multisite study and the differences in methodology. Furthermore, aggregation of data may also attenuate the prevalence of certain geographical areas.

Our study shows a lower proportion of HPV in laryngeal carcinoma (9.1%) compared to other studies that used P16 IHC. Hernandez et al. in their laryngeal cancer-focused study found a 12.5% HPV positivity [10]. Ahmed et al. and Faggons et al. found 26 and 33% prevalences respectively [15, 16]. Sekee et al. utilised P16 and PCR and found a prevalence of 13.9 and 5.06% respectively, further explaining how different methods of detecting HPV can give varying results [17]. Laryngeal cancers are mostly attributed to tobacco use in a dose- dependent manner with little evidence of involvement of high risk HPV types [4, 10]. In our study, more than half of our participants smoked.

The prevalence of HPV in hypopharyngeal carcinoma (14.3%) in our study was lower than that found by Yang et al. (26.1%) in China that similarly used P16 IHC [11]. This higher proportion may be due to research on only hypopharyngeal tumors increasing their sample size. Sekee et al. found a prevalence of 20% positivity with a strong agreement between PCR and p16 IHC for the hypopharyngeal carcinomas [17]. Their study findings agree with our observation probably due to geographical similarities. Faggons et al. found no HPV in hypopharyngeal cancers whereas Ahmed et al. aggregated the oropharynx and hypopharynx into pharynx limiting our use of their findings [15, 16].

### HPV positive verses HPV negative

We secondarily compared HPV status in the UADT SCC with gender, age, substance use and sexual habits and did not find any statistical significance.

All our participants were black with a male predominance (a male to female ratio for HPV positive of 3:1 and HPV negative of 4.2: 1.) Several studies similarly found the majority of patients with HPV related cancer to be male [13, 22]. The age at presentation was skewed towards the elderly with the most common age category as 51-60 years for both HPV positive and HPV negative groups similar to that of Sekee et al. [17].

Of the HPV positive group, 41% used both alcohol and tobacco and the same percentage did not use any of the mentioned substances. In contrast, in the HPV negative group, 23% neither smoked nor took alcohol while 55% used both alcohol and tobacco. These findings suggest a higher chance of HPV positivity in non- substance users as shown in Herero’s study [22].

In our study, participants under 40 years were all HPV negative, with majority being HIV negative non- substance users. This would suggest another pathway that may not be viral, probably genetic or dietary and poses an opportunity for further research in our setting.

Concerning sexual behavior, the trends for lifetime sexual partners and oral sexual habits in both groups were similar. Oral sexual practices were low and 18% declined to answer questions on oral sexual habits. This was not surprising as it is generally considered a taboo to talk about or disclose sexual practices in most of our cultural settings.

This study was done at Uganda Cancer Institute which receives patient referrals from across the country so the findings of this study may be generalizable to Uganda. We were able to get histology samples within a year of diagnosis increasing our chances of viability in the tissues and the slides were read by two consultant pathologists further strengthening the reliability of the results.

P16 is a surrogate marker and this limits the findings of our study since we were unable to confirm them by PCR or ISH. Furthermore, it was beyond the scope of this study to infer correlation between P16 status (denoting HPV) and age, gender, substance use and sexual habits.

## Conclusion

The prevalence of HPV using P16 IHC in UADT SCC at UCI was significant at 20.3%. The commonest site affected by HPV was the oropharynx closely followed by the oral cavity.

Particular attention with routine screening of patients with SCC involving the oropharynx/ oral cavity should be taken. We recommend studies using ISH and PCR to confirm our findings with P16 IHC.

## Supplementary information

**Additional file 1.** Data Collection Form.

**Additional file 2.** Standard Harris’ Haematoxylin and Eosin stain for Paraffin sections (Clayden, 1971).

**Additional file 3.** P16 Staining.

**Additional file 4.** Scoring of P16 gene expression immuno-staining.

**Additional file 5.** AMERICAN JOINT COMMITTEE ON CANCER (AJCC).

## Data Availability

The datasets used and/or analyzed during the current study are available from the corresponding author on request.

## Abbreviations

DNA: Deoxyribo Nucleic Acid
ENT: Ear Nose Throat
FFPE: Formalin Fixed Paraffin Embedded
H&E: Haematoxylin and Eosin
HIV: Human Immunodeficiency Virus
HN: Head and Neck
HNC: Head and Neck Carcinoma
HNSCC: Head and Neck Squamous Cell Carcinoma
HPV: Human Papilloma Virus
NOP: Non-Oropharyngeal
OP: Oropharyngeal
OPSCC: Oro-pharyngeal Squamous Cell Carcinoma
P16 IHC: P16 Immunohistochemistry
SCC: Squamous Cell Carcinoma
SOP: Standard Operating Procedure
SSA: Sub Saharan Africa
UADT: Upper Aero-digestive Tract
UADT SCC: Upper Aero-digestive Tract Squamous Cell Carcinoma
UCI: Uganda Cancer Institute
USA: United States of America
