# Supplementary material for: A descriptive study of human papilloma virus in upper aero-digestive squamous cell carcinoma at Uganda cancer institute assessed by P16 immunohistochemistry
Source: Cancers Head Neck. 2020 Aug 27;5:10. doi: 10.1186/s41199-020-00057-3 (PMC7450959; doi:10.1186/s41199-020-00057-3)
Supplement: Supplementary file 1 — Additional file 1. Data Collection Form. [file 41199_2020_57_MOESM1_ESM.pdf]

Appendix I: Data Collection Form

Unique identification number ………………………………

Date of interview …………………………………

Name of researcher ………………………………….

A. Particulars of patient clinical presentation

1. Age …………………………………………………

2. Gender a) Male b) female

3. Address ………………………………………………

4. Occupation

a) Civil servant b) subsistence farmer c) business d) unemployed

5. Monthly income

a) < 100,000/= b) 100,000-500,000/= c) > 500, 000/=

6. Highest level of education

a) None b) Primary c) Secondary d) Tertiary e) Vocational

7. Have you ever used tobacco products?

a) Yes b) No

8. If yes, for how long?

a) < 5years b) 5-10years c) 11-20 years d) >20 years

9. What product do you use?

a) Chewing tobacco b) cigarettes c) Pipe

10. Have you ever used alcohol?

a) Yes b) No

11. If yes, tick the appropriate answe

a) Regular daily b) regular weekly c) occasionally

12. HIV status (documented)

a) Negative b) positive

13. How many sexual partners have you had in your lifetime?

a) None b) One c) more than one d) declined to answer

14. Have you ever engaged in oral sex?

a) Yes b) No c) declined to answer

15. Tumor site

a) Oral cavity b) oropharynx c) larynx d) Hypopharynx

16. Tumor size

a) T1 b) T2 c) T3 d) T4

17. Node size

a) N0 b) N1 c) N2 d) N3

18. Metastasis

a) M0 b) M1

19. Tumor stage

a)Stage 0 b)Stage I c)Stage II d)Stage III e)Stage IV

20. Histological grade of tumor by H&E (put a tick against correct description)

a) Well differentiated b) Moderately differentiated c) Poorly differentiated d)Other (specify)

21. p16 expression

a)Positive b) Negative
