# Supplementary material for: A descriptive study of human papilloma virus in upper aero-digestive squamous cell carcinoma at Uganda cancer institute assessed by P16 immunohistochemistry
Source: Cancers Head Neck. 2020 Aug 27;5:10. doi: 10.1186/s41199-020-00057-3 (PMC7450959; doi:10.1186/s41199-020-00057-3)
Supplement: Supplementary file 2 — Additional file 2. Standard Harris’ Haematoxylin and Eosin stain for Paraffin sections (Clayden, 1971). [file 41199_2020_57_MOESM2_ESM.pdf]

Appendix II

Standard Harris' Haematoxylin and Eosin stain for Paraffin sections (Clayden, 1971)

Method:

1. Sections were de-waxed through grades of xylene and hydrated alcohols (concentrations from 100%, 5%, 90%, 75% and to 70%) to water.
2. They were then stained in Haematoxylin for 5-15 minutes.
3. They were washed well in running tap water until the sections "blued" for at least 5 minutes.
4. The sections were then differentiated in 1% acid alcohol (1% HCL in 70% alcohol) for 5-10 seconds.
5. Sections were washed in running tap water and left to "blue" for 10-15 minutes.
6. Sections were counterstained with Eosin for 5-10 minutes.
7. They were then washed in running tap water for 1-5 minutes.
8. Sections were then dehydrated through increased concentrations of alcohol for a few seconds.
9. They were then washed in running tap water for 1-5 minutes.
10. Finally, the sections were cleared in two changes of xylene and mounted under the cover slips using DPX.
