# Supplementary material for: A descriptive study of human papilloma virus in upper aero-digestive squamous cell carcinoma at Uganda cancer institute assessed by P16 immunohistochemistry
Source: Cancers Head Neck. 2020 Aug 27;5:10. doi: 10.1186/s41199-020-00057-3 (PMC7450959; doi:10.1186/s41199-020-00057-3)
Supplement: Supplementary file 3 — Additional file 3. P16 Staining. [file 41199_2020_57_MOESM3_ESM.pdf]

Appendix III: P16 Staining

Positive control as recommended by the manufacturer; uterine cervical cancer tissue with greater than 80% staining for p16 protein was used as a positive control in parallel with the test tissue sections. Negative control used was human brain tissue.

P16 staining protocol

1. The paraffin embedded tissue was de-paraffinised in the oven at 30 degrees for 15 minutes.

2. Sections were de-waxed by passage through xylene and rehydrated by a graded series of ethanol, and brought to water.

3. Each section was then washed in flex water buffer for 10 minutes.

4. The sections were then incubated in Flex retrieval solution diluted 1:50 at 90 degrees Celsius in a de-clocking chamber.

5. Endogenous peroxidase was then activated by 3% hydrogen peroxide in phosphate- buffered saline (PBS, pH 7.4) for 10 minutes.

6. Each section was then rinsed in Flex wash buffer for 10 minutes.

7. The primary antibody p16 was then applied at a dilution of 1 in 200 microlitres and incubated in the humidity chamber for 20 minutes.

8. The sections were then carefully washed in flex wash buffer for 10 minutes.

9. Excess water was bloated out and secondary antibody was applied and the section incubated in the humidity chamber for 10 minutes.

10. The section was then washed in flex wash buffer for 5 minutes.

11. Horse radish peroxidase was then applied on the section and left in situ for 5 minutes.

12. Visualization was then performed with Diaminobenzidinetetra hydrochloride mixed in substrate buffer.

13. Sections was then rinsed in flex wash buffer and sections were counterstained with Haematoxylin for one minute.

14. Then the sections were blued in water, dehydrated in absolute ethanol.

15. Clearance was done in xylene and the sections were cover slipped.

16. Nuclear staining and cytoplasmic staining were considered positive for p16 expression
