# Supplementary material for: A descriptive study of human papilloma virus in upper aero-digestive squamous cell carcinoma at Uganda cancer institute assessed by P16 immunohistochemistry
Source: Cancers Head Neck. 2020 Aug 27;5:10. doi: 10.1186/s41199-020-00057-3 (PMC7450959; doi:10.1186/s41199-020-00057-3)
Supplement: Supplementary file 4 — Additional file 4. Scoring of P16 gene expression immuno-staining. [file 41199_2020_57_MOESM4_ESM.pdf]

Appendix IV: Scoring of P16 gene expression immuno-staining.

Positive results were reported with regard to:

Site of staining: as either nucleus or cytoplasm

Intensity of staining: graded from 0(negative) to grade 3(strong)

Percentage of tumor cells staining: at (x40) was graded from grade 0 (negative) to grade IV.

Immuno-reactivity of P16 protein expression was graded into four groups according to the fraction of positive tumor cells and intensity of positive of positive staining.

*Grade III: strong/high immuno-reactivity in more than 25% of tumor cells.*

*Grade II: moderate immuno-reactivity in more than 25% of tumor cells*

*Or strong immuno-reactivity in less than 25% of the tumor cells.*

*Grade I: Low/ weak immuno-reactivity in more than 25 % of the tumor cells*

*Or Moderate immuno-reactivity in less than 25% of the tumor cells.*

*Grade 0: lack of immuno-staining (negative)*

*Or weak immuno-staining in less than 25% of the tumor cells*

Grade II and III were considered positive whereas grades 0 and I as normal tissue.
