# Supplementary material for: A descriptive study of human papilloma virus in upper aero-digestive squamous cell carcinoma at Uganda cancer institute assessed by P16 immunohistochemistry
Source: Cancers Head Neck. 2020 Aug 27;5:10. doi: 10.1186/s41199-020-00057-3 (PMC7450959; doi:10.1186/s41199-020-00057-3)
Supplement: Supplementary file 5 — Additional file 5. AMERICAN JOINT COMMITTEE ON CANCER (AJCC). [file 41199_2020_57_MOESM5_ESM.pdf]

Appendix V: AMERICAN JOINT COMMITTEE ON CANCER (AJCC)

TNM (TUMOUR, NODE, METASTASIS) STAGING MANUAL 2014.

Primary Tumour of Oral cavity (T)

Includes: oral tongue, buccal mucosa, hard palate, alveolar ridge, retromolar trigone, floor of mouth

T1 2 cm or less in greatest dimension

T2 > 2 cm but < 4 cm in greatest dimension

T3 > 4 cm in greatest dimension

T4a Moderately advanced local disease*

Tumor invades through cortical bone, inferior alveolar nerve, floor of mouth, or skin of face—that is, chin or nose (oral cavity).

Tumor invades adjacent structures (e.g., through cortical bone, into deep extrinsic muscle of tongue, maxillary sinus, skin of face)

T4b Very advanced local disease

Tumor invades masticator space, pterygoid plates, or skull base and/or encases internal carotid artery

*NB: Superficial erosion alone of bone/tooth socket by gingival primary is not sufficient to classify as T4.

Primary Tumor of Oropharynx (T)

Includes: base of tongue, inferior surface of the soft palate and uvula, anterior and posterior tonsillar pillars, glossotonsillar sulci, pharyngeal tonsils, lateral and posterior pharyngeal walls

T1 2 cm or less in greatest dimension

T2 > 2cm and < 4cm in greatest dimension

T3 > 4 cm in greatest dimension, extension to lingual surface of epiglottis

T4a Moderately advanced local disease.

Invades larynx, extrinsic tongue muscles, medial pterygoid, hard palate, or mandible*

T4b Very advanced local disease.

Invades lateral pterygoid muscle, pterygoid plates, lateral nasopharynx or skull base, or encases carotid artery

*NB: Mucosal extension to lingual surface of epiglottis from primary tumors of the base of the tongue and vallecula does not constitute invasion of larynx.

Primary Tumour of Hypopharynx (T)

Includes: the pyriform sinuses, the lateral and posterior hypopharyngeal walls, and the postcricoid region.

T1 Tumor limited to one subsite of the hypopharynx

2 cm or less in greatest dimension

T2 Tumor invades more than one subsite of the hypopharynx or an adjacent site

> 2 cm but < 4 cm in greatest dimension

without fixation of the hemilarynx or extension to the esophagus

T3 > 4 cm in greatest dimension

or with fixation of the hemilarynx or extension to the esophagus

T4a Moderately advanced local disease, invades thyroid/cricoid cartilage, hyoid bone,

thyroid gland, esophagus, or central compartment soft tissues

T4b Very advanced local disease

Tumor invades prevertebral fascia, encases carotid artery, or involves mediastinum

Primary Tumor of Larynx (T)

Divided into:

Supraglottis -lingual and laryngeal and infrahyoid epiglottis, false cords, arytenoids, aryepiglottic folds

Glottis -True vocal folds(anterior and posterior commissures) occupies a horizontal place 1 cm in thickness, extending inferiorly from the lateral margin of the ventricle

Subglottis - extends from the lower boundary of the glottis to the lower margin of the cricoid cartilage

Supraglottis

T1 limited to one subsite of the supraglottis with normal vocal fold mobility

T2 Invades mucosa of more than one adjacent subsite of the supraglottis or glottis or

region outside the supraglottis (e.g., mucosa of base of tongue, vallecula,

medial wall of pyriform sinus) without fixation of the larynx

T3 Tumor limited to the larynx with vocal fold fixation and/or

invades any of the following: postcricoid area, pre-epiglottic tissues, paraglottic

space, and/or inner cortex of thyroid cartilage

T4a Moderately advanced local disease

invades through the thyroid cartilage and/or tissues beyond the larynx e.g.trachea, deep extrinsic muscle of the tongue, strap muscles, thyroid, or esophagus)

T4b Very advanced local disease

invades prevertebral space, encases carotid artery, or invades mediastinal structures

Glottis

T1 Tumor limited to the vocal fold(s)

(may involve anterior or posterior commissure) with normal mobility

T1a Tumor limited to one vocal fold

T1b Tumor involves both vocal folds

T2 Tumor extends to the supraglottis and/or subglottis,

and/or with impaired vocal fold mobility

T3 Tumor limited to the larynx with vocal fold fixation and/or

invasion of paraglottic space, and/or inner cortex of the thyroid cartilage

T4a Moderately advanced local disease

Tumor invades the outer cortex of the thyroid cartilage and/or tissues beyond the larynx

T4b Very advanced local disease

invades prevertebral space, encases carotid artery, or invades mediastinal structures

Subglottis

T1 Tumor limited to the subglottis

T2 Tumor extends to the vocal cord(s) with normal or impaired mobility.

T3 Tumor limited to the larynx with vocal fold fixation.

T4a Moderately advanced local disease

Tumor invades cricoid or thyroid cartilage and/or invades tissues beyond the larynx

T4b Very advanced local disease

invades prevertebral space, encases carotid artery, or invades mediastinum.

Nodal staging for regional lymph nodes (N)

N0	No nodes
N1	Ipsilateral < 3 cm
N2a	Ipsilateral > 3 cm and < 6 cm
N2b	Ipsilateral multiple < 6 cm
N2c	Bilateral or contralateral < 6 cm
N3	> 6 cm

Distant metastases (M)

MX Distant metastasis cannot be assessed

M0 No distant metastasis

M1 Distant metastasis

Stage grouping

Oral cavity, oropharynx, hypopharynx, larynx

Stage I	TI N0 M0
Stage II	T2 N0 M0
Stage III	T3 N0 M0
	T1-3 N1 M0
Stage IV	T4 N0 M0
	Any T N2 M0
	Any T N3 M0
	Any T Any N M1
